# *Thinopyrum ponticum* Chromatin-Integrated Wheat Genome Shows Salt-Tolerance at Germination Stage

**DOI:** 10.3390/ijms16034512

**Published:** 2015-02-26

**Authors:** Wen-Ye Yuan, Motonori Tomita

**Affiliations:** 1 Research Institute of Green Science and Technology, Shizuoka University, 836, Ohya, Suruga-ku, Shizuoka 422-8529, Japan; 2 Molecular Genetics Laboratory, Faculty of Agriculture, Tottori University, 101, Minami 4-chome, Koyama-cho, Tottori 680-8553, Japan

**Keywords:** wheat, *Thinopyrum ponticum*, salt tolerance, dominant gene block, translocation, genomic *in situ* hybridization (GISH)

## Abstract

A wild wheatgrass, *Thinopyrum ponticum* (2*n* = 10*x* = 70), which exhibits substantially higher levels of salt tolerance than cultivated wheat, was employed to transfer its salt tolerance to common wheat by means of wide hybridization. A highly salt-tolerant wheat line S148 (2*n* = 42) was obtained from the BC_3_F_2_ progenies between *Triticum aestivum* (2*n* = 42) and *Th. ponticum*. In the cross of S148 × salt-sensitive wheat variety Chinese Spring, the BC_4_F_2_ seeds at germination stage segregated into a ratio of 3 salt tolerant to 1 salt sensitive, indicating that the salt tolerance was conferred by a dominant gene block. Genomic *in situ* hybridization analysis revealed that S148 had a single pair of *Th. ponticum*–*T. aestivum* translocated chromosomes bearing the salt-tolerance. This is an initial step of molecular breeding for salt-tolerant wheat.

## 1. Introduction

Salinity is a major external factor affecting plant growth and productivity. Of the world’s 230 million ha of all irrigated lands, 19.5% are seriously affected by salinity (http://www.fao.org/nr/land/en/). Wheat is grown under irrigated and rain-fed conditions; both types of agriculture are threatened by salinization [1,2]. Therefore, the development of salt-tolerant wheat is an important breeding objective. To date, however, little progress has been made towards improving the salt tolerance of wheat. Shalaby *et al.* [3] indicated that breeding salt tolerant wheat by exploiting naturally occurring variation is difficult.

Fortunately, amongst halophytic members of Triticeae, tall wheatgrass species (e.g., *Thinopyrum* spp.) have received the most attention as a potential gene source for improving salt tolerance in wheat [4,5]. A perennial grass, *Th. ponticum* (Podp.) Barkworth and D.R.Dewey (2*n* = 10*x* = 70, JJJJsJs) was used as forage on salt-affected lands [6]. Several accessions of *Th. ponticum* survived 750 mM NaCl [7] and some maintained reasonable growth at an EC of 13.9 dS·m^–1^ [8]. Thus, *Th. ponticum* is a promising material to transfer its salt tolerance into common wheat by chromosome engineering through wide hybridization. Towards the improvement of salt tolerance of wheat, wheat-tall wheatgrass amphiploids was developed as a new salt-tolerant cereal, named *Tritipyrum* (derived from *Triticum* spp. × *Thinopyrum* spp.) [9,10,11]. Although the resulting crop would not be expected to produce grain quality of bread or durum wheat, it could likely be used as feed wheat.

The second step is to develop recombinant wheat lines containing small segments of tall wheatgrass chromosomes without linkage drag, such as deleterious effects on yield or grain quality. In this study, *Th. ponticum* chromosomes was transferred to *T. aestivum* by back crossing onto the amphiploids of *T. aestivum* × *Th. ponticum*, and that the salt-tolerance small chromatin was successfully inherited at germination stage in the BC_4_F_2_.

## 2. Results and Discussion

### 2.1. Transfer of Th. ponticum Genetic Material with Salt Tolerance to Wheat by Wide Hybridization

Firstly, an amphiploid TAe68 (2*n* = 56) developed from the hybrid of *T. aestivum* salt-sensitive variety Jinmai 24 × *Th. ponticum* showed salinity tolerance. TAe68 was backcrossed to “Jinmai 24” twice. Until the BC_2_ crossing, the hybrid embryo was excised 15–20 days after pollination and cultured on the media for callus induction and then plantlets were differentiated. Through the BC_2_F_2_ to BC_2_F_6_ generation, plants grown in a greenhouse were selected according to the salt tolerance test. Chromosome numbers were examined using 2% (*w*/*v*) aceto-carmine, and *Th. ponticum* chromatins were identified by C-banding analysis according to Gill *et al.* [12]. In the BC_2_F_6_ generation, a highly salt-tolerance with 2*n* = 42 was selected and backcrossed twice with *T. aestivum* salt-sensitive cv. Chinese Spring. The BC_4_F_1_ line showed 21 bivalents in pollen mother cells. These results indicated that BC_3_F_2_ line S148 is a *Th. ponticum*–*T. aestivum* translocation line.

**Figure 1:**
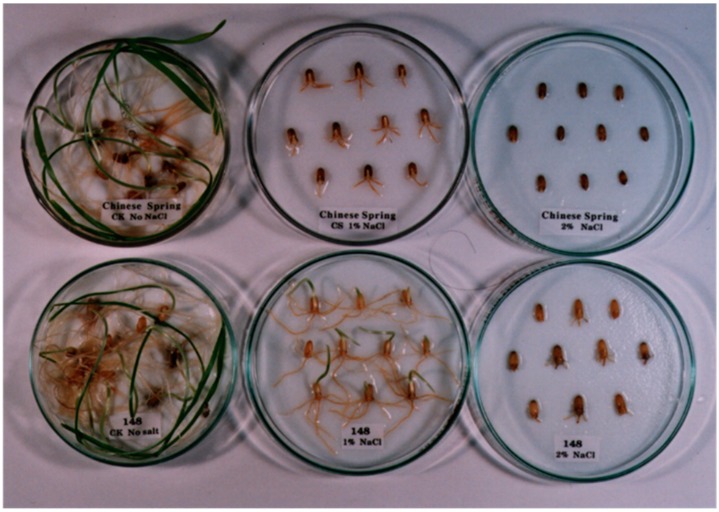
Salt tolerance test of Chinese Spring (**above**) and S148 (**below**) at the germination stage. A, B, and C: NaCl conc. 0.0, 0.2 and 0.4 M, respectively.

Grains of a salt-resistant line S148, Chinese Spring, and the BC_4_F_2_ progeny of Chinese Spring × S148 were used for genetic analysis for salt tolerance under NaCl conc. 0.1, 0.2, 0.3 and 0.4 M, respectively. Both genotypes germinated in the presence of 0.1 and 0.2 M NaCl, but subsequent coleoptile and root extension was retarded. Notably, only S148 was able to germinate in the presence of 0.4 M NaCl and showed the least reductions in coleoptile and root lengths (Figure 1). In the cross of S148 × Chinese Spring, the BC_4_F_2_ seeds segregated into a ratio of 72 salt tolerant to 27 salt sensitive. In salt tolerant plants, the averaged coleoptile and root length were 17.5 and 21.4 mm, respectively. Whereas, in salt sensitive plants, averaged coleoptile and root length were 3.5 and 6.8 mm, respectively. This segregation ratio was well fitted to a ratio of 3:1 (χ^2^ = 0.273; 0.50 < *p* < 0.75), indicating that the salt tolerance was conferred by a dominant gene block derived from *Th. ponticum.*

**Figure 2:**
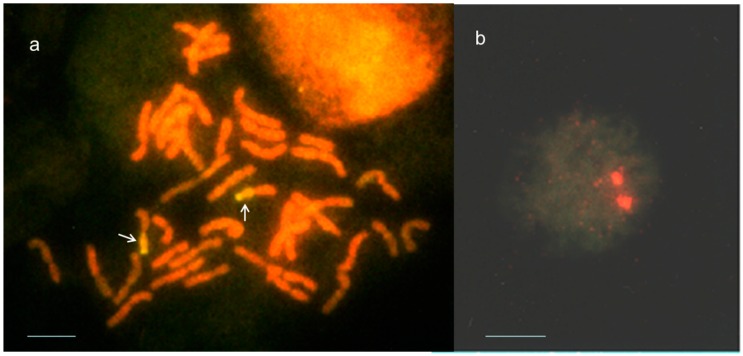
Genomic *in situ* hybridization (GISH) results of line S148. (**a**) Two translocated chromosome segments (arrows) originated from *Th. ponticum* were visualized in a mitotic metaphase cell; (**b**) Two fluorochrome signals of *Th. ponticum* were detected in a mitotic interphase cell. Bars represent 10 μm.

### 2.2. Identification of Th. ponticum Chromatin in Salt-Tolerant Recombinant Wheat

Fluorescence GISH method according to Tomita *et al.* [13] was applied to detect the *Th. ponticum* chromatin in the wheat background. The total genomic DNA of *Th. ponticum* labeled with biotin-16-dUTP or Digoxigenin-11-dUTP was hybridized as the probe to the denatured chromosomes. In the double-exposed photographs, the *in situ* hybridization sites on *Th. ponticum* chromosome segments were visualized using FITC or rhodamine, and the other chromosomes were counter-stained with PI or DAPI (Figure 2). Using this approach, two alien chromosome segments were detected in S148 and some salt resistant individuals in BC_4_F_2_. GISH analysis verified that S148 had a single pair of *Th. ponticum*–*T. aestivum* translocated chromosomes including the salt tolerance gene block of *Th. ponticum*. The observed single genotypic ratio of salt tolerance means that this alien translocation transmitted normally and did not have segregation distortion.

Soil salinity is a major abiotic constraint to agricultural productivity. Introgression of genes conferring salt tolerance from *Th. ponticum* (2*n* = 10*x* = 70) into *Triticum aestivum* is one of the few methods by which salt-tolerant wheat varieties can be developed. The modern breeding strategy is developing recombinant lines of wheat containing small segments of tall wheatgrass chromosomes without deleterious effects on yield or grain quality. Such an approach has enabled the use of tall wheatgrass to confer disease resistance to wheat [14,15,16,17,18]. However, *Th. ponticum*–*T. aestivum* translocation lines with the salt tolerance have not been developed, and the salt tolerance of *Th. ponticum* has not been employed in wheat varieties. In this study, we attempted to identify alien chromatin in the progenies resulting from *T. aestivum*–*Th. ponticum* hybrids using genomic *in situ* hybridization method. Here, salt tolerance of *Th. ponticum* had been introgressed into bread wheat as a stably inherited translocated chromatin. This study is an initial step to developing the salt tolerant wheat plant. The future studies for line S148 will be further characterized by molecular cytogenetic approaches to reveal the specific chromosome arms of wheat and tallgrass which are involved to the translocation, as in the case of wheat-*Lophopyrum elongatum* recombinant lines [19]. Molecular genetic analysis of salt tolerance is also important [18,20]. Salt tolerance in decaploid tall wheatgrass was associated with a capacity to restrict the rate of accumulation of sodium ion. Obtained *Th. ponticum*–*T. aestivum* translocation line with salt stress tolerance could potentially lead to identification of salt tolerance determinants.

## 3. Experimental Section

### 3.1. Embryo Culture

The culture media for callus induction and callus maintenance are: (1) Murashige and Skoog (MS) medium + 200 mg/L glutamine + 100 mg/L asparagine + 600 mg/L hydrolytic lacto-albumin + 2 mg/L 2,4-D + 0.1 mg/L KT; (2) MS + 1 mg/L 2,4-D + 1 mg/L NAA + 0.1 mg/L KT; and (3) MS + 2 mg/L 2,4-D. The culture medium for differentiation is MS + 3 mg/L BA. Sugar 3%, agar 0.6%, pH = 5.6. Culture was maintained under a photoperiod of 14 h/day and temperature of 25 ± 1 °C.

### 3.2. Salinity Test

The seeds were sown in Petri dishes on filter paper soaked with distilled water and 0.1, 0.2, 0.3 and 0.4 M NaCl and the dishes were placed in an incubator at 25 °C. After 7 days, coleoptile and main root lengths were measured. Electroconductivity was measured daily and the salinity was adjusted as needed to maintain the NaCl.

### 3.3. Fluorescence Genomic in Situ Hybridization

The total genomic DNA of *Th. ponticum* was labeled with biotin-16-dUTP or Digoxigenin-11-dUTP (Roche Diagnostics, Basel, Switzerland), and was hybridized as the probe to the denatured chromosomes. After hybridization, biotin-labeled probe was detected by avidin-fluorescein isothiocyanate (FITC) with signal amplification using a biotinylated anti-avidin secondary antibody and fluorescein-avidin DCS (a cell sorter grade of fluorescein avidin D) (Roche Diagnostics). The Digoxigenen-labeled probe was detected by anti-Dig-rhodamine (Roche Diagnostics). Chromosomes were excited using a WBV or WIG filters (Olympus, Tokyo, Japan) and counter-staining with propidium iodide (PI) or 4',6-Diamidine-2'-phenylindole dihydrochloride (DAPI) (Roche Diagnostics).

## References

[1] Ghassemi F., Jakeman A.J., Nix H.A. (1995). Salinization of land and water resources. Human Causes, Extent, Management and Case Studies.

[2] Mujeeb-Kazi A., Diaz de Leon J.L., Ahmad R., Malik K.A. (2002). Conventional and alien genetic diversity for salt tolerant wheats: Focus on current status and new germplasm development. Prospects for Saline Agriculture.

[3] Shalaby E.E., Epstein E., Qualset C.O. (1993). Variation in salt tolerance among some wheat and triticale genotypes. J. Agron. Crop Sci..

[4] Colmer T.D., Munns R., Flowers T.J. (2005). Improving salt tolerance of wheat and barley: Future prospects. Aust. J. Exp. Agric..

[5] Wang R.R.C., Li X.M., Hu Z.M., Zhang J.Y., Larson S.R., Zhang X.Y., Grieve C.M., Shannon M.C. (2003). Development of salinity-tolerant wheat recombinant lines from a wheat disomic addition line carrying a *Thinopyrum junceum* chromosome. Int. J. Plant Sci..

[6] Colmer T.D., Flowers T.J., Munns R. (2006). Use of wild relatives to improve salt tolerance in wheat. J. Exp. Bot..

[7] McGuire G.E., Dvořák J. (1981). High salt tolerance potential in wheatgrasses. Crop Sci..

[8] Dewey D.R. (1960). Salt tolerance of twenty-five strains of *Agropyron*. Agron. J..

[9] Akhtar J., Gorham J., Qureshi R.H. (1994). Combined effect of salinity and hypoxia in wheat (*Triticum aestivum* L) and wheat-*Thinopyrum* amphiploids. Plant Soil.

[10] King I.P., Law C.N., Cant K.A., Orford S.E., Reader S.M., Miller T.E. (1997). *Tritipyrum*, a potential new salt-tolerant cereal. Plant Breed..

[11] Brasileiro-Vidal A.C., Cuadrado A., Brammer S.P., Benko-Iseppon A.M., Guerra M. (2005). Molecular cytogenetic characterization of parental genomes in the partial amphidiploid *Triticum aestivum* × *Thinopyrum ponticum*. Genet. Mol. Biol..

[12] Gill B.S., Friebe B., Endo T.R. (1991). Standard karyotype and nomenclature system for description of chromosome bands and structural aberrations in wheat (*Triticum aestivum*). Genome.

[13] Tomita M., Shinohara K., Morimoto M. (2008). *Revolver* is a new class of transposon-like gene composing the Triticeae genome. DNA Res..

[14] Pienaar R.V. (1990). Wheat × *Thinopyrum* hybrids. Biotechnology in Agriculture and Forestry.

[15] Fedak G. (1999). Molecular aids for integration of alien chromatin through wide crosses. Genome.

[16] Chen S.Y., Xia G.M., Quan T.Y., Xiang F.N., Yan J., Chen H.M. (2004). Introgression of salt-tolerance from somatic hybrids between common wheat and *Thinopyrum ponticum*. Plant Sci..

[17] Wang J., Xiang F.N., Xia G.M. (2005). *Agropyron elongatum* chromatin localization on the wheat chromosomes in an introgression line. Planta.

[18] Wang M.C., Peng Z.Y., Li C.L., Li F., Liu C., Xia G.M. (2008). Proteomic analysis on a high salt tolerance introgression strain of *Triticum aestivum*/*Thinopyrum ponticum*. Proteomics.

[19] Mullan D.J., Mirzaghaderi G., Walker E., Colmer T.D., Francki M.G. (2009). Development of wheat-*Lophopyrum elongatum* recombinant lines for enhanced sodium “exclusion” during salinity stress. Theor. Appl. Genet..

[20] Mott I.W., Wang R.R.C. (2007). Comparative transcriptome analysis of salt-tolerant wheat germplasm lines using wheat genome arrays. Plant Sci..

